# *Pseudomonas aeruginosa* biofilm killing beyond the spacer by antibiotic-loaded calcium sulfate beads: an in vitro study

**DOI:** 10.5194/jbji-6-119-2021

**Published:** 2021-03-23

**Authors:** Jacob R. Brooks, Devendra H. Dusane, Kelly Moore, Tripti Gupta, Craig Delury, Sean S. Aiken, Phillip A. Laycock, Anne C. Sullivan, Jeffrey F. Granger, Matthew V. Dipane, Edward J. McPherson, Paul Stoodley

**Affiliations:** 1 Department of Microbial Infection and Immunity, The Ohio State University, Wexner Medical Center, Columbus, Ohio, USA; 2 Biocomposites Ltd., Keele Science Park, Keele, Staffordshire, ST5 5NL, UK; 3 Department of Orthopaedics, The Ohio State University, Wexner Medical Center, Columbus, Ohio, USA; 4 Department of Orthopaedic Surgery, David Geffen School of Medicine at UCLA, Santa Monica, California, USA; 5 National Centre for Advanced Tribology at Southampton (nCATS), National Biofilm Innovation Centre (NBIC), Department of Mechanical Engineering, University of Southampton, Southampton, UK

## Abstract

**Introduction**: Bacterial biofilms are an important virulence factor in chronic periprosthetic joint infection (PJI) and other orthopedic infection since they are highly tolerant to antibiotics and host immunity. Antibiotics are mixed into carriers such as bone cement and calcium sulfate bone void fillers to achieve sustained high concentrations of antibiotics required to more effectively manage biofilm infections through local release. The effect of antibiotic diffusion from antibiotic-loaded calcium sulfate beads (ALCS-B) in combination with PMMA bone cement spacers on the spread and killing of *Pseudomonas aeruginosa* Xen41 (PA-Xen41) biofilm was investigated using a “large agar plate” model scaled for clinical relevance. **Methods**: Bioluminescent PA-Xen41 biofilms grown on discs of various orthopedic materials were placed within a large agar plate containing a PMMA full-size mock “spacer” unloaded or loaded with vancomycin and tobramycin, with or without ALCS-B. The amount of biofilm spread and log reduction on discs at varying distances from the spacer was assessed by bioluminescent imaging and viable cell counts. **Results**: For the unloaded spacer control, PA-Xen41 spread from the biofilm to cover the entire plate. The loaded spacer generated a 3 cm zone of inhibition and significantly reduced biofilm bacteria on the discs immediately adjacent to the spacer but low or zero reductions on those further away. The combination of ALCS-B and a loaded PMMA spacer greatly reduced bacterial spread and resulted in significantly greater biofilm reductions on discs at all distances from the spacer. **Discussion**: The addition of ALCS-B to an antibiotic-loaded spacer mimic increased the area of antibiotic coverage and efficacy against biofilm, suggesting that a combination of these depots may provide greater physical antibiotic coverage and more effective dead space management, particularly in zones where the spread of antibiotic is limited by diffusion (zones with little or no fluid motion).

## 1 Introduction

Periprosthetic joint infection (PJI) is a complex problem in total joint arthroplasty (TJA) and occurs in 2 %–2.4 % of all total hip and knee replacement procedures (Kurtz et al., 2012; Rasouli et al., 2014). While infrequent, PJI has a dramatic effect on the patient's health, often resulting in joint dysfunction, morbidity, and mortality (Vrgoc et al., 2014; Boddapati et al., 2018; Zmistowski et al., 2013). The financial burden placed on patients and the healthcare system is staggering (Kurtz et al., 2012; Kamath et al., 2015). A major complicating factor in treating PJI is microbiota-produced biofilms (Gbejuade et al., 2015; McConoughey et al., 2014). Biofilms are pathogenic communities that adhere to living and nonliving surfaces and exhibit greatly increased antibiotic tolerance and resistance against host immunity. The establishment of biofilms is assisted by the presence of foreign materials of orthopedic implant components, such as various metals and polymers (Zimmerli, 2014; Moley et al., 2019).

*Pseudomonas aeruginosa* is a Gram-negative opportunistic nosocomial pathogen and is cultured up to 20 % of the time in chronic Gram-negative PJI (McConoughey et al., 2014; Zmistowski et al., 2011; Rodríguez-Pardo et al., 2014). In previous in vitro studies, *P. aeruginosa* has shown the ability to display tolerance and resistance to antibiotics commonly used to treat PJIs (Dusane et al., 2019). Although the Gram-positive Staphylococci are the most commonly isolated pathogens from PJI, the infecting organism (or organisms) may not be cultured as much as up to 25 % of the time (Kapadia et al., 2016; Pulido et al., 2008; Choi et al., 2013) or treatment is started before culture data are available. Thus, in treating PJI, combinations of antibiotics are commonly used to provide broad-spectrum coverage (Ciofu et al., 2017). Vancomycin and/or aminoglycosides (most commonly gentamicin or tobramycin) can be mixed into polymethyl methacrylate (PMMA) bone cement and mineral-based absorbable bone fillers such as calcium sulfate dihydrate (CaSO${}_{4}$ ⋅2 H${}_{2}$O) to be administered as spacers (Hansen et al., 2014) and beads (McPherson et al., 2013) in the joint space and medullary canals. These depot forms have been shown to release high concentrations of antibiotic required to more effectively prevent or manage biofilms that cannot be achieved by systemic administration (Mandell et al., 2019). There is promising yet inconclusive clinical data suggesting that ALCS-B used as an adjuvant in revision arthroplasty result in more favorable outcomes (Abosala and Ali, 2020). However, it is not known how ALCS-B used in combination with antibiotic-loaded PMMA may impact the area of antimicrobial potency against biofilms, particularly in those areas such as dead zones where there is little fluid flow, and the spread of the antibiotic is limited by diffusion.

Previous studies using in vitro biofilms of the bioluminescent *P. aeruginosa* Xen41 (PA-Xen41) showed that the number and spacing of ALCS-B were important in the rate and extent of killing of an agar lawn biofilm (Dusane et al., 2019). Further, we have previously shown in a similar model that the antibiotic loading concentration in ALCS-B did not significantly change the effective area of antibiotic activity over the observed time frame, from which we concluded that the area of antibiotic potency was limited primarily by diffusion, not the loading potency (Dusane et al., 2017). While we expect that increasing the area of spread of antibiotic depots will result in increased spatial coverage of antibiotic activity, we wish to determine the spatial effect of adding ALCS-B to an antibiotic-loaded spacer mimic on the spread of bacteria from biofilm-colonized materials, as well as the killing efficacy of biofilm bacteria on those materials in a scaled-up in vitro model. In the present study, we evaluate the effect of ALCS-B and antibiotic-loaded bone cement spacers (ALBC-S) on containing both the spread and killing of *P. aeruginosa* biofilms using a “large agar plate” model. We used *P. aeruginosa* Xen41 in part because of its strong bioluminescent signal, allowing for more sensitive and illustrative monitoring of the effect of antibiotic diffusing from the PMMA or ALSC-B by an in vitro imaging system (IVIS).

## 2 Materials and methods

### 2.1 Bacterial growth

A bioluminescent derivative strain of *Pseudomonas aeruginosa* PAO1 (PA-Xen41; Xenogen Corp., USA) was used. Stock culture was streaked onto 1.5 % tryptic soy agar (TSA; Becton, Dickinson & Company, MD, USA) and incubated for 24 h. A single colony was transferred aseptically to 15 mL of tryptic soy broth (TSB; Becton, Dickinson & Company, MD, USA) and incubated overnight at 37 ${}^{\circ }$C, 5 % CO${}_{2}$ on a rotary shaker at 200 rpm. We used PA-Xen41 for our studies because it gives off a very strong signal, allowing for our long-term non-destructive monitoring of the spread of antibiotics on activity and killing of the lawn biofilms, and as mentioned previously, *P. aeruginosa* is a relevant Gram-negative PJI pathogen.

### 2.2 Formation of biofilms on circular discs

Overnight cultures were diluted to 0.1 % and used to inoculate sterile, circular discs (BioSurface Technologies, MT, USA) of hydroxyapatite (HA), ultra-high molecular weight polyethylene (UHMWPE), 316L stainless steel (SS-316), and titanium (Ti). The “as received” roughnesses measured by contact profilometry were 976, 3867, 224, and 300 nm, respectively. The discs had a diameter of 9.5 mm and a thickness of 2 mm. Four milliliters of the diluted culture was added to four different wells of a six-well plate and three discs of each material were aseptically submerged in the inoculum. The plate was incubated at 37 ${}^{\circ }$C, 5 % CO${}_{2}$ for 72 h to establish 3 d biofilms.

### 2.3 Preparation of antibiotic-loaded calcium sulfate beads (ALCS-B)

ALCS-B were prepared using Stimulan^®^ Rapid Cure (Biocomposites, Ltd., Keele, UK) 10-cc mixing kits. Twenty grams of CaSO${}_{4}$ powder was mixed with 1000 mg of vancomycin hydrochloride powder (VAN; Tokyo Chemical Industry, Tokyo, Japan) and 240 mg of tobramycin sulfate powder (TOB; VWR International, PA). Once blended, 6 mL of sterile liquid included in the kit was added and the mixture stirred into a uniform paste for 30 s. The paste was pressed into a flexible mold (Biocomposites, Ltd., Keele, UK) with bead sizes of 4.8 mm in diameter and allowed to set for 10–15 min at 20 ${}^{\circ }$C. Although Xen41 is resistant to vancomycin (Dusane et al., 2017), we used a combination of vancomycin and tobramycin since the combination of vancomycin with an aminoglycoside (tobramycin or gentamicin) has been mixed in PMMA and ALCS-B clinically to provide broad-spectrum coverage (Anagnostakos, 2017; Hanssen and Spangehl, 2004). Additionally, we wanted to be consistent with previous experiments using ALCS-B (McConoughey et al., 2015, 2014; Dusane et al., 2017; Howlin et al., 2017) and avoid changing the release characteristics and set time by changing the loading formulation.

### 2.4 Preparation of antibiotic-loaded (LS) and unloaded (US) PMMA bone cement spacer mimics

PMMA bone cement spacer mimics were prepared using Simplex™ P SpeedSet™ Radiopaque Bone Cement construction kits (Stryker, Kalamazoo, MI), which are commonly used clinically. Antibiotic-loaded spacers were fabricated using 2 g VAN powder, 2 g TOB powder, and 40 g PMMA powder. Unloaded spacers were prepared with only PMMA bone cement powder. Twenty milliliters of sterile methyl methacrylate monomer (liquid) was added to the powder mixture, stirred into a dough-like mass, formed into a “hockey puck-like” disc using a 7 cm diameter circular mold (Silikomart Professional Silicone Baking Mold, Cylinder 6 Cavities, Amazon, WA) and allowed to set for 30 min at 20 ${}^{\circ }$C.

**Figure 1:**
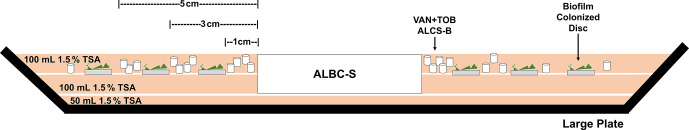
Side-view schematic showing the construction of the large plate agar model. This model allows for the spread of antibiotic released from ALCS-B and an antibiotic-loaded spacer (ALBC-S) as it diffuses through the agar. Three discs of each material containing 3 d biofilms were placed at distances of 1, 3, and 5 cm radiating linearly from the edge of the PMMA spacer mimic. ALCS-B were only included in the LS + ALCS-B antibiotic condition.

### 2.5 Large plate model

Fifty milliliters of 1.5 % TSA was added to a 21 cm diameter glass pie baking dish sterilized with 70 % EtOH and allowed to solidify, followed by central placement of a PMMA spacer followed by another 100 mL of molten agar. Once set, three discs of each material (12 in total, each colonized with pre-grown 3 d biofilms) were placed on top of the agar layer radiating outwards (Fig. 1). For the experiments with ALCS-B, a 10-cc pack of beads was sprinkled somewhat randomly around the spacer with greater bead density closer to the spacer mimic. While sprinkling the ALCS-B without careful placement in a specified pattern may cause difficulty with interpretation, we wished to more closely mimic how they might be applied clinically, where they might not be spread evenly. Also, small diameter antibiotic depots such as ALCS-B can fill smaller void spaces than the larger PMMA beads and thus can be distributed more thoroughly.

After adding the beads, another 100 mL of cooled (∼ 50 ${}^{\circ }$C) liquid agar was poured to cover the discs and beads and the plate covered with plastic film wrap for incubation and imaging. The antibiotic-loaded spacer and biofilm-colonized discs were embedded within layers of agar to allow for the diffusion of antibiotic and bacteria from all surfaces. These agar “layers” merged to form one cohesive block (Fig. 1). The following conditions were tested: (1) unloaded PMMA spacer only (US), (2) a VAN + TOB antibiotic-loaded spacer only (LS), and (3) a VAN + TOB-loaded spacer with a 10-cc pack of ALCS-B (LS + ALCS-B). The plates were incubated at 37 ${}^{\circ }$C, 5 % CO${}_{2}$ for 5 d and imaged daily. Previously we demonstrated that the spreads of tobramycin as a function of time (t) over a 4 d period to achieve killing of the Xen41 agar lawn biofilms from PMMA and ALCS beads were 4.2 t${}^{0.5}$ mm and 3.6 t${}^{0.5}$ mm respectively, with the loading concentration making little difference, suggesting the transport through the agar was limited by diffusion (Dusane et al., 2017).

### 2.6 Bioluminescent imaging (BLI)

BLI was executed using an in vitro imaging system (IVIS 100, Xenogen, MA) that semi-quantitatively measures the relative amount of metabolically active biofilm. Each quadrant of the large plate model was individually imaged and then stitched together (Photoshop, Adobe, CA) to show the whole plate. Red represented the highest metabolic activity and blue or black low or no metabolic activity. White-light (plain) images of each plate were captured with a cellphone.

### 2.7 Viable cell counting

Colony-forming unit (CFU) counts were performed on mimicked sets of discs, one set on the 3 d biofilms and one set after incubation in the large plate model. CFUs were performed as previously described (Moley et al., 2018). These discs were rinsed and then vortexed with 10 mL of phosphate-buffered saline (PBS; Dulbecco's, Gibco, Thermo Fisher Scientific, MA) in 15 mL Falcon tubes (Thermo Fisher Scientific, MA); 10 µL of each dilution of a 10-fold dilution series was spotted onto TSA. The plates were incubated at 37 ${}^{\circ }$C, 5 % CO${}_{2}$ for 24 h and colonies enumerated to determine CFU per area of disc (CFU/cm${}^{2}$). CFU counts after the incubation were done by first extracting the embedded discs as an “agar plug” in which a 1.35 cm diameter circular glass tube was used to punch out the coupons.

### 2.8 Statistical analysis

All experiments were performed in triplicate. The effect of the different conditions on the log reduction of biofilm at different proximities from the spacer was compared by first performing a log${}_{10}$ transformation and the geometric means used to calculate log reductions. Our CFU detection limit was 3.5 log${}_{10}$ CFU/cm${}^{2}$. Discs that displayed no CFU growth are shown as equal to or less than this limit. The log reductions of the LS and LS + ALCS-B antibiotic conditions were compared by a Student's t test assuming unequal variances, where p<0.05 was considered statistically significant. Data were analyzed and graphed using Excel software (version 2102, Microsoft 365).

## 3 Results

### 3.1 Prevention of biofilm spread by LS and ALCS-B

**Figure 2:**
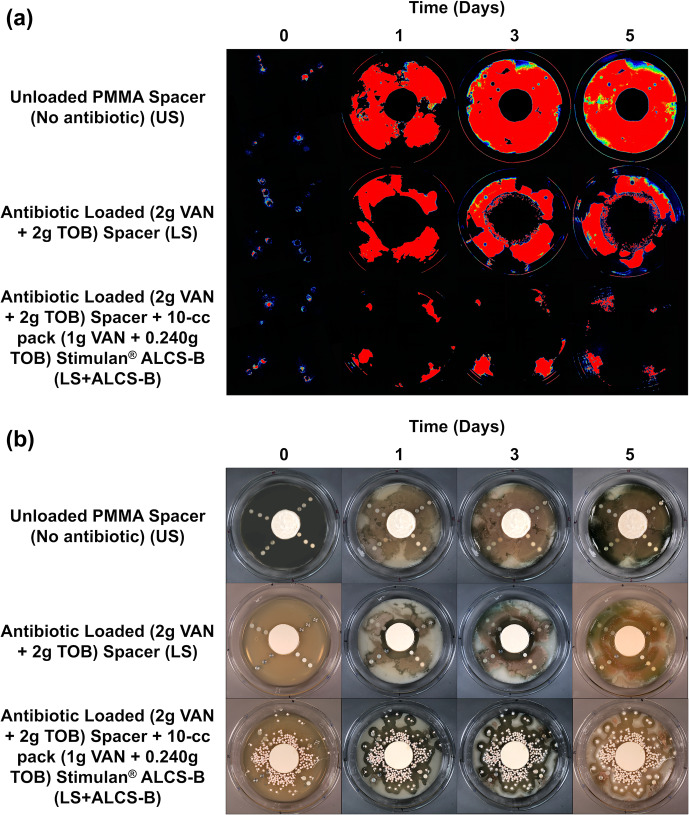
IVIS **(a)** and white-light **(b)** images tracking the suppression of *P. aeruginosa* Xen41 biofilm spread from biofilm-colonized discs of four different orthopedic materials for three different antibiotic conditions. Plates were imaged every 24 h for 5 d. At the periphery of all plates, there is a loss of bioluminescence over time, even in the non-antibiotic control. This is likely due to nutrient depletion and a loss of metabolic activity in this region.

The bioluminescence of *P. aeruginosa* Xen41 allowed biofilm on the discs and the spread of bacteria from these discs to be easily visualized while remaining in situ in the large plate model over the 5 d (Fig. 2a). The unloaded spacer (US) condition of the large plate, containing no antibiotic, showed substantial bacterial spread from the discs and was confirmed by white-light imaging, showing *P. aeruginosa* coverage throughout the large plate (Fig. 2b).

The antibiotic-loaded spacer (LS) alone exhibited anti-bacterial activity generating a zone of inhibition (ZOI) of approximately 2.4–3.0 cm from the edge of the spacer (Fig. 2a). After 3 d a small number of colonies were observed growing a few millimeters within the edge ZOI (Fig. 2a). These were possibly antibiotic tolerant phenotypes and have been observed previously (Dusane et al., 2019).

The LS + ALCS-B condition, which contained the addition of VAN + TOB ALCS-B, prevented the formation of antibiotic-tolerant phenotypes and suppressed the spreading of PA-Xen41 to sparse areas near the peripheral edge of the plate, where the bead density was lowest, or in areas where beads were absent (Fig. 2).

**Figure 3:**
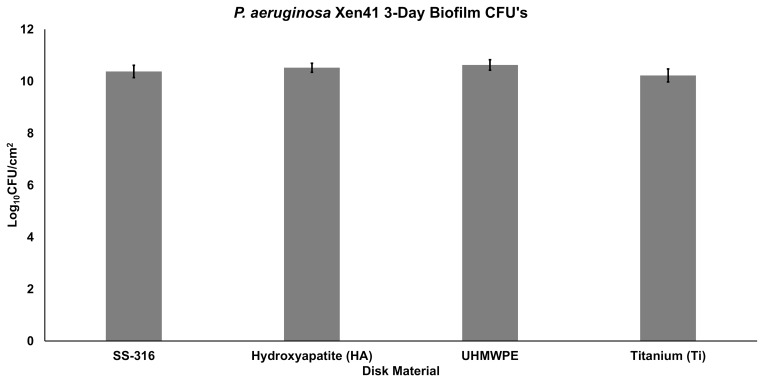
Quantification of viable bacteria (by CFU) on discs containing *P. aeruginosa* Xen41 3 d biofilm. Counts are reported in terms of log${}_{10}$ CFUs per surface area of the disc (in cm${}^{2}$).

### 3.2 Region of biofilm killing by viable cell counts

After 3 d of inoculation, the amount of PA-Xen41 biofilm grown on various discs was quantified by cell counting (CFU). This analysis was completed to confirm biofilm growth on each type of disc before implementation into the large plate model. All four disc materials displayed sizeable biofilm growth of $∼10^{10}$CFU/cm${}^{2}$, although there was no statistically significant difference in growth between any of the four materials (p>0.05). UHMWPE discs garnered the largest number of bacteria with a count of 10.6 log${}_{10}$ CFU/cm${}^{2}$ (Fig. 3), followed by HA, SS-316, and Ti, indicating a weak trend with increasing roughness.

**Figure 4:**
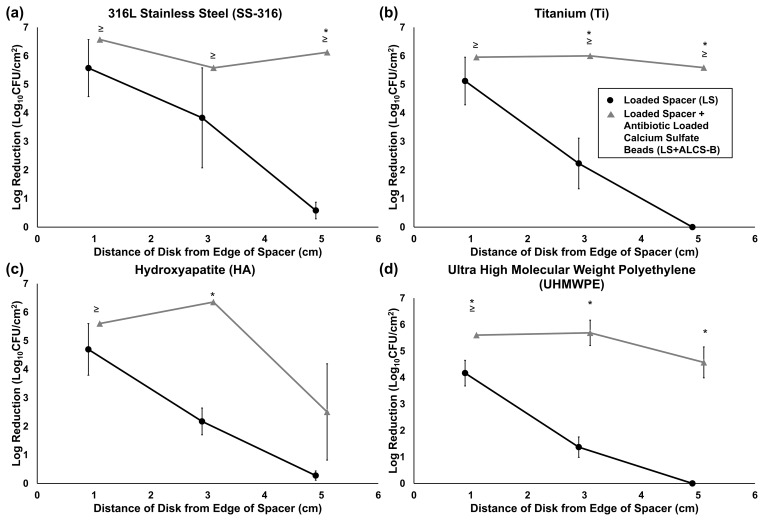
Log reduction of *P. aeruginosa* Xen41 biofilms, after being grown for 3 d in TSB media and then introduced into the large plate model against one of three antibiotic conditions for 5 d. CFU counts of SS-316 **(a)**, Ti **(b)**, HA **(c)**, and UHMWPE **(d)** from the biofilm-colonized discs, varying in distance from the edge of the PMMA spacer, were compared to those on an unloaded spacer (US) control. ≥ denotes discs eradicated of biofilm bacteria to below the CFU detection limit (3.54 log${}_{10}$ CFU/cm${}^{2}$) and indicates that the reduction could be greater than or equal to the value listed in the graph. * represents statistically significant (p<0.05) differences in log reductions between LS and LS + ALCS-B antibiotic conditions.

The LS alone reduced biofilm concentrations on the closest discs (1 cm from the spacer's edge), with bacterial reduction ranging from 1.5 to 5.5 log${}_{10}$ CFU/cm${}^{2}$ depending on the material type (Fig. 4a–d). For the more distant discs (3 and 5 cm from the spacer), reduction of bacteria drastically decreased to a range of no reduction to 3.8 log${}_{10}$ CFU/cm${}^{2}$. The addition of loaded beads (LS + ALCS-B) promoted the log reduction of the more distant discs to levels of 2.5 to 6.6 log${}_{10}$ CFU/cm${}^{2}$, depending on the material. Furthermore, the addition of ALCS-B eradicated biofilm to below detection limits (3.5 log${}_{10}$ CFU/cm${}^{2}$) on SS-316 and Ti discs 5 cm from the spacer (Fig. 4a, b), a statistically significant reduction compared to the LS alone. In UHMWPE (Fig. 4d), the LS + ALCS-B condition produced significantly more (p<0.05) biofilm reduction in discs at every distance. The Ti discs 3 and 5 cm from the edge of the spacer (Fig. 4b), the HA disc 3 cm from the spacer (Fig. 4c), and the SS-316 disc 5 cm from the spacer (Fig. 4a) also showed statistically significant biofilm killing in the LS + ALCS-B condition compared to the LS alone (p<0.05).

## 4 Discussion

Here we show in our diffusion-based model that ALCS-B significantly reduced (p<0.05) *P. aeruginosa* Xen41 biofilm growth on materials common to orthopedic implants in vitro while also increasing the zone of antimicrobial potency beyond that achieved by an antibiotic-loaded PMMA spacer alone. Furthermore, our work demonstrates how such beads sprinkled around the spacer may more effectively manage dead space and control the spread of bacteria from such implant surfaces, particularly in those locations where the spread of released antibiotics is limited by diffusion, as would be expected in the periprosthetic tissue, areas of a joint space, or the medullary canal. In a well-mixed system, the specific distribution of antibiotic depots will be less important since the antibiotics will be well distributed by the mixing, but when the rate of spread of antibiotics is limited by diffusion, then the spatial distribution becomes significant.

The observed spread of bacteria from the discs over the surrounding agar appears similar to how biofilm is thought to spread throughout a joint space infection (Zimmerli and Sendi, 2017; Jenkins et al., 2010). Antibiotics loaded into PMMA cement in the form of both beads and spacers have been successfully used to treat PJI for many years (Hanssen and Spangehl, 2004). However, there are limitations to the use of PMMA alone with respect to the release of local antibiotics at a surgical site. Once an antibiotic has eluted to below MIC levels, the PMMA itself becomes a surface for bacterial biofilm formation (Ma et al., 2018; Stoodley et al., 2008). Further, even though retrieved PMMA spacers are still shown to elute antibiotics and produce a ZOI in laboratory testing, the infection can persist, suggesting that the zone of antimicrobial potency may be limited (Swearingen et al., 2016).

Antibiotic-loaded PMMA beads have been used to increase the effective area of antibiotic activity, but they have limited penetration into small spaces and require surgical removal. Additionally, their relatively large size (∼1 cm diameter) limits the number that can be packed into a given volume. In contrast, ALCS-B allow for the rapid, full release of antibiotics (Dusane et al., 2017), and our visual demonstration of ZOI produced by loaded PMMA spacers alone, compared to spacers with ALCS-B, indicates how ALCS-B can aid in releasing antibiotics to areas beyond the spacer.

We also note that since the beads in our study were deliberately not positioned carefully but rather scattered semi-randomly, ALCS-B either ended up relatively close to one or more of the distant (3 and 5 cm) discs or fell farther away. This resulted in variability of killing efficacy. If beads fell close to a biofilm-colonized disc, then there was a significant log reduction or eradication to below-detection limits, while discs further away from beads exhibited less or no reduction. This spatial variation is evidenced by some of the large error bars (Fig. 4a–d) and illustrates the importance of adequate spatial coverage within a periprosthetic joint or other infected surgical sites. Nevertheless, our in vitro data as well as those found clinically (Morgenstern et al., 2018; Ciofu et al., 2017), and data from other in vitro studies (Howlin et al., 2015, 2017; Laycock et al., 2018; Knecht et al., 2018) support the use of ALCS-B as an effective means of releasing antibiotic to prevent biofilm spread and kill pre-grown bacterial biofilms.

We recognize limitations in our study. Our study was conducted with only one bacterial strain. While we have shown similar efficacy with other Gram-negative and -positive species with respect to prevention and killing efficacy by antibiotics released from beads alone (Howlin et al., 2017, 2015), to add rigor to our conclusions, we would need to include more pathogenic species and clinical strains. In addition, we used a relatively young biofilm grown for 3 d which may be less antibiotic-tolerant and less clinically relevant than more mature biofilms. Also, our in vitro model is simplistic compared to the complex mechanical and chemical environment of surrounding bones, particularly that of an articulating human joint space. By design our model lacks any fluid flow, which would be expected to enhance the spread of antibiotic from the spacer mimic and the ALCS-B while also removing antibiotic from the system (i.e., there is no infinite sink where antibiotic can be removed from the system). A pharmacokinetic study by Hayes et al. (2014) found the intra-articular concentration of gentamicin released from a sponge in a dog dropped rapidly from approximately 2400 to 4 µg/mL after 22.4 h. They concluded that this drop was due to vascular exchange and inflammation. However, conventional in vitro studies of elution kinetics from antibiotic-loaded depots are also oversimplified. In these studies, the depot is placed in a reservoir of saline and the concentration measured periodically. In some cases there is periodic complete or partial exchange of the saline to create an infinite sink, where the flux of the released drug is not limited by its build-up in the reservoir (Fu and Kao, 2010). While these established kinetic studies are useful for directly comparing the release kinetics of different materials and indeed have been used to demonstrate faster release from ALCS-B than from PMMA beads (McConoughey et al., 2015), these are completely mixed systems and there are no gradients other than in the mass boundary layer at the depot surface. Thus, they provide no information about how the spatial arrangement of such depots may influence the local distribution of antibiotics. Our diffusion model represents the opposite extreme of a completely mixed system, yet we argue it has value for illustrating how antibiotics may spread from depots when transport is limited by diffusion and how the size, number, and spatial arrangement of such depots may influence such spread. In a healthy knee, the flow of synovial fluid caused by flexion of the joint during cyclic loading cycles is thought to provide enough convective transport to deliver nutrients to periarticular tissues, which can be up to 1 cm away from blood vessels (Levick, 1995). Such a mechanism is also expected to distribute antibiotics eluted from local carriers or delivered systemically from the vasculature. However, it is not well understood how well this transport mechanism functions in a reconstructed, revised, and potentially immobilized joint. Radical debridement of the PJI joint has been shown to compromise local blood flow to regions of the affected joint, especially bone. While such debridement may be necessary to rid the infected area of biofilm to the best extent possible, when vasculature is compromised, the spread of antibiotics through bone and joint tissues may be expected to be largely limited by diffusion (Thabit et al., 2019). Other complicating factors such as diabetes, kidney disease, or osteonecrosis secondary to osteomyelitis may also limit the flow of systemic fluids. Diffusion is a much slower process, particularly over longer distances, making ALCS-B a potentially useful adjunct to antibiotic-loaded bone cement spacers (or other forms) for distributing the necessary amount of antibiotics in a timely and effective manner. It is also possible that the lack of flow may have resulted in pH changes or high calcium levels in the vicinity of the beads. In previous studies we have demonstrated that unloaded beads alone do not have antibacterial activity (McConoughey et al., 2015), though we did not investigate antibiotic–local chemistry interactions.

The results from our in vitro study may support the adjuvant use of ALCS-B in the management of PJI, particularly for the release of antibiotics to the interstices of the periprosthetic joint space in light of limited PMMA diffusion with compromised vasculature. However, further work is required in order to determine how our in vitro findings relate to the spread of antibiotics in the human joint or may relate to clinical outcomes. Such studies are not trivial since measuring antibiotic concentrations at different locations in tissues and fluids in a joint space requires multiple samplings at different times, since sampling of joint fluid may not represent conditions everywhere bacteria or biofilm may be present. We are currently developing a continuous flow reactor system which can accommodate clinically relevant antibiotic-loaded spacer mimics and beads, where we match the flow rate with reported clinical drainage rates following TKA revision. Such a system may provide further insight into the complex interplay between antibiotic release kinetics from various administration methods, including addition of antibiotic powder.

## Data Availability

Raw data used to generate the graphs in Figs. 3 and 4 and showing the statistical analysis available by request or download from DOI (Stoodley and Brooks, 2021).

## Appendix A Abbreviations

**Table:**

PJI	Periprosthetic joint infection
TJA	Total joint arthroplasty
PMMA	Polymethyl methacrylate
ALBC-S	Antibiotic-loaded bone cement spacer
ALCS-B	Antibiotic-loaded calcium sulfate beads
PA-Xen41	*Pseudomonas aeruginosa* Xen41
TSA	Tryptic soy agar
TSB	Tryptic soy broth
HA	Hydroxyapatite
UHMWPE	Ultra-high molecular weight polyethylene
SS-316	316L stainless steel
Ti	Titanium
VAN	Vancomycin
TOB	Tobramycin
US	Unloaded spacer
LS	Loaded spacer
LS + ALCS-B	Loaded spacer and antibiotic-loaded calcium sulfate beads
BLI	Bioluminescent imaging
IVIS	In vitro imaging system
CFUs	Colony-forming units
ZOI	Zone of inhibition
